# Emerging roles of non‐coding RNAs in scoliosis

**DOI:** 10.1111/cpr.12736

**Published:** 2019-12-12

**Authors:** Zheng Li, Xingye Li, Jianxiong Shen, Lin Zhang, Matthew T. V. Chan, William K. K. Wu

**Affiliations:** ^1^ Department of Orthopaedic Surgery Peking Union Medical College Hospital Chinese Academy of Medical Sciences and Peking Union Medical College Beijing China; ^2^ Department of Orthopedic Surgery Beijing Jishuitan Hospital Fourth Clinical College of Peking University Jishuitan Orthopaedic College of Tsinghua University Beijing China; ^3^ Department of Anaesthesia and Intensive Care The Chinese University of Hong Kong Hong Kong Hong Kong; ^4^ State Key Laboratory of Digestive Diseases Centre for Gut Microbiota Research Institute of Digestive Diseases and LKS Institute of Health Sciences The Chinese University of Hong Kong Hong Kong Hong Kong

**Keywords:** circRNAs, lncRNAs, miRNAs, non‐coding RNAs, scoliosis

## Abstract

Scoliosis, a complex three‐dimensional deformity of the spine with the Cobb angle (a measure of the spinal lateral curvature) >10 degree, encompasses a spectrum of pathologies, including congenital, idiopathic, syndromic and neuromuscular aetiologies. The pathogenesis is multifactorial involving both environmental and genetic factors but the exact cellular and molecular mechanisms of disease development remain largely unknown. Emerging evidence showed that non‐coding RNAs (ncRNAs), namely microRNAs, long ncRNAs and circular RNAs, are deregulated in many orthopaedic diseases, including scoliosis. Importantly, these deregulated ncRNAs functionally participate in the initiation and progression of scoliosis. Here, we review recent progress in ncRNA research on scoliosis.

## 1 INTRODUCTION

Scoliosis is defined as an abnormal lateral curvature of the spine with the Cobb angle >10 degree and inevitably accompanied by varying degree of hyperlordosis and rotational deformity.^1^, ^2^, ^3^ Scoliosis has several subtypes including idiopathic, syndromic, neuromuscular and congenital scoliosis, among which adolescent idiopathic scoliosis (AIS) is the most common and affects about 1%‐4% of adolescents around the world.^4^, ^5^ Congenital spinal malformation occurring during embryogenesis leading to mixed segmental vertebral deformity is another common cause of scoliosis.^6^ The aetiologies of scoliosis are multifactorial, involving both environmental (eg, maternal alcohol use and vitamin deficiency during pregnancy for congenital scoliosis) and genetic (eg single‐nucleotide polymorphisms [SNPs] in *LBX1*, *GPR126*, *BNC2* and *PAX1* for idiopathic scoliosis) factors.^7^, ^8^, ^9^, ^10^, ^11^, ^12^, ^13^, ^14^, ^15^, ^16^ However, the exact cellular and molecular mechanisms linking these aetiological factors to the development of scoliosis remain largely unknown. Thus, it is crucial to study the molecular pathogenesis of scoliosis so as to identify novel molecular markers for early identification of patients at risk and the development of mechanism‐driven therapeutics.

Non‐coding RNAs (ncRNAs) are an important class of regulatory, non‐protein‐coding transcripts, which encompass three major subclasses, namely long ncRNAs (lncRNAs), microRNAs (miRNAs) and circular RNAs (circRNAs).^17^, ^18^, ^19^, ^20^ While the mechanism by which miRNAs regulate gene expression is relatively straightforward (ie, guiding the RNA‐induced silencing complex to target mRNAs through base‐pairing to induce their degradation and/or inhibit their translation),^21^ lncRNAs and circRNAs could regulate gene expression at multiple levels (eg, DNA methylation, histone modification, recruitment of transcriptional factors, sponging of miRNAs and regulation of mRNA stability for lncRNAs; sponging of miRNAs, transcription regulation, modulation of alternative splicing, direct interactions with RNA‐binding proteins and protein translation via rolling circle amplification for circRNAs).^22^, ^23^ ncRNAs are essential for the regulation and execution of key cellular processes, including proliferation, apoptosis, autophagy, differentiation, metabolism, migration, differentiation and invasion.^24^, ^25^, ^26^, ^27^, ^28^, ^29^, ^30^ It therefore comes as no surprise that ncRNAs are deregulated in most, if not all, kinds of diseases, including neoplastic, inflammatory and metabolic diseases.^31^, ^32^, ^33^, ^34^, ^35^ From a clinical perspective, the frequent alteration of ncRNA abundance in the body fluid, such as saliva, blood and urine, during diseases make this class of molecules promising candidates for development into biomarkers for early diagnosis and prognostication.^36^, ^37^, ^38^

A growing body of evidence now suggests that deregulated expression of ncRNAs is a key to the development of orthopaedic diseases, including osteosarcoma, osteoporosis, osteoarthritis and intervertebral disc degeneration.^25^, ^28^, ^39^, ^40^, ^41^ Emerging evidence also suggests that ncRNAs are deregulated in scoliosis and functionally participate in its pathogenesis.^42^, ^43^ Here, we first summarize recent ncRNA profiling studies in scoliosis followed by discussing specific ncRNAs that are functionally important to the development of scoliosis. The potential clinical utilities of ncRNAs as molecular biomarkers and therapeutic targets for the management of scoliosis are also addressed.

## 2 ncRNA EXPRESSION PROFILING IN SCOLIOSIS

Profiling the expression of ncRNAs with whole‐transcriptome sequencing, microarray or PCR array followed by confirmation with reverse transcription (RT)‐quantitative PCR is the most common approach to identify and validate deregulated ncRNAs in specific disease states.^17^, ^44^, ^45^ In this regard, multiple groups of investigators have embarked on profiling studies to depict the transcriptome‐wide expression landscape of ncRNAs in scoliosis (Table 1).

**Table 1:** ncRNAs expression profiles in scoliosis

No.	Method	Sample	Microarray filtering criteria	Deregulated	Upregulated	Downregulated	References
1	Microarray, RT‐PCR	AIS cases healthy children	fold change more than 2 and *P* < .05	546 mRNAs 139 lncRNAs	512 mRNAs 118 lncRNAs TCONS00028768 ENST00000440778.1 NR024075	34 mRNAs ENST00000414894.1 ENST00000440778.1	42
2	Microarray, RT‐PCR	AIS cases healthy children	*P* < .05; Fold change > 2		miR‐1226‐5p, miR‐27a‐5p, miR‐223‐5p, miR‐122‐5p	miR‐671‐5p, miR‐1306‐3p	44
3	Microarray, RT‐PCR	Patients with Friedreich's ataxia	*P* < .05; Fold change > 1.5	miR‐128‐3p, miR‐625‐3p, miR‐130b‐5p, miR‐151a‐5p, miR‐330‐3p, miR‐323a‐3p, miR‐142‐3p, miR‐16‐5p			48
4	Transcriptome sequencing, RT‐PCR	Degenerate disc tissues	Fold change > 2	749 mRNAs, 70 circRNAs, 685 lncRNAs, 56 miRNAs	194 mRNAs, 185 lncRNAs, 35 circRNAs, 53 miRNAs	555 mRNAs, 500 lncRNAs, 35 circRNAs, 3 miRNAs	43

Liu and colleagues performed Agilent mRNA and lncRNA human Array V3.0 microarray to identify the expression patterns of mRNA and lncRNA in peripheral blood of healthy children and AIS cases.^46^ Their data demonstrated that a total of 546 mRNAs and 139 lncRNAs were differentially expressed between healthy control and AIS cases, among which NR024075 and ENST00000440778.1 were the most upregulated and downregulated lncRNAs, respectively. RT‐qPCR confirmed the significant upregulation of ENST00000414894.1, TCONS 00028768 and ENST00000602322.1 and downregulation of ENST000004407‐78.1 in AIS. Among the 546 differentially expressed mRNAs, 34 mRNAs were downregulated in the AIS group, while 512 mRNAs were overexpressed. Furthermore, the correlations between lncRNA expression levels and clinical features of scoliosis were explored. The investigators found that the expression levels of TCONS00028768 and ENST00000440778.1 were correlated with the height of scolitic patients. Patients with higher ENST00000602322.1 expression levels were also younger at onset. Lower expression of ENST00000414894.1 was observed in scoliotic patients with Cobb angles more than 40 degrees while higher expression of ENST00000440778.1 was correlated with higher Risser grade.

Efforts have also been put forth to profile circulating miRNAs in patients with scoliosis. García‐Giménez and colleagues profiled circulating miRNAs by RNA sequencing in 17 AIS patients and 10 healthy controls, followed by validation with RT‐qPCR in 30 AIS patients and 13 healthy controls.^47^ Their data suggested that circulating miRNAs of AIS patients exhibited differential abundance profiles as compared with healthy subjects. miR‐122‐5p, miR‐27a‐5p and miR‐223‐5p were found to be more abundant in patients with AIS. These 3 miRNAs together with another miRNA miR‐1306‐3p as a combined miRNA signature could differentiate AIS patients from controls with an area under the curve (AUC) value of 0.95. These data suggested that circulating miRNAs may be used as potential diagnostic biomarkers for AIS. In another study, Seco‐Cervera and colleagues profiled circulating miRNAs by RNA sequencing in 17 controls and 25 patients with Friedreich's ataxia (FRDA), which is an autosomal recessive neurodegenerative mitochondrial disease with non‐neurological features including scoliosis, cardiac complications and diabetes.^24^, ^48^ The differential abundance of miR‐128‐3p, miR‐625‐3p, miR‐130b‐5p, miR‐151a‐5p, miR‐330‐3p, miR‐323a‐3p, miR‐142‐3p, and miR‐16‐5p was further confirmed by RT‐qPCR. Nevertheless, differentially abundant circulating miRNAs identified in the studies by García‐Giménez et al and Seco‐Cervera et al did not overlap, which might be explained by the aetiological heterogeneity of scoliosis.

Besides using human samples for ncRNA profiling, differential expression of ncRNAs pertinent to the pathogenesis of scoliosis can be inferred from related animal models. Our previous study used whole‐transcriptome sequencing to determine ncRNA and mRNA expression profiles in rat embryos with vitamin A deficiency (VAD)‐induced congenital scoliosis as compared to control.^42^ A total of 56 miRNAs, 70 circRNAs, 685 lncRNAs and 749 mRNAs were found to be significantly differentially expressed between the two groups, among which there were 555 downregulated and 194 upregulated mRNAs; 500 downregulated and 185 upregulated lncRNAs; 35 downregulated and 35 upregulated circRNAs; and 3 downregulated and 53 upregulated miRNAs in the VAD embryos. Bioinformatic analysis is the involvements of forkhead box O (FoxO), phosphatidylinositol 3‐kinase (PI3K)‐Akt, mammalian target of rapamycin (mTOR), epidermal growth factor receptor (EGFR) and Wnt signalling pathways in the pathogenesis of VAD‐induced congenital scoliosis. The deregulation of selected ncRNAs (miRNAs: miR‐187‐5p and miR‐466c‐3p; lncRNAs: NONRATG027649.1 and NONRATG024332.1; circRNAs: chr5_50556456_51183813 and chr15_23792823_23793342) was confirmed with RT‐qPCR.

These profiling studies, mostly performed with peripheral blood of patients, collectively suggested that ncRNAs are deregulated in scoliosis. It is expected that the use of other tissue types, such as bone, paravertebral muscle and cartilage, collected during surgery for transcriptome‐wide profiling will depict a much clearer picture of ncRNA deregulation in a cell‐type‐specific manner that will guide the choice of cell and animal models for subsequent functional studies.

## 3 FUNCTIONAL ROLES OF KEY ncRNAS IN SCOLIOSIS

### 3.1 miR‐4300

Ogura and colleagues performed the genome‐wide association study (GWAS) on 2142 patients with AIS, among which 1105 exhibited clinical progression while the remaining 832 were assigned to the non‐progression group.^49^ After replicating the association of top 10 candidate loci using an independent cohort of AIS patients (323 progressed and 283 non‐progressed patients), strong association of chromosome 11q14.1 containing the SNP rs1828853 with AIS progression was identified. SNPs exhibiting strong linkage disequilibrium with rs1828853 were found to be clustered in putative enhancer region in intron 1 of *MIR4300HG*, which codes for miR‐4300. Among SNPs in linkage disequilibrium with rs1828853, only rs35333564 showed the allelic difference in the binding to the nuclear proteins, with the risk allele associated with a decrease in enhancer activity. These data suggest that rs1828853 could alter miR‐4300 expression to dictate the risk of AIS progression.

### 3.2 miR‐145

Disturbance of bone turnover in patients is implicated in the pathogenesis of AIS. Zhang and their colleagues used miRNA microarray to profile miRNA expression in bone tissues of iliac crest from AIS patients compared to control subjects and identified miR‐145 as an AIS‐associated miRNA.^50^ RT‐qPCR confirmed the aberrant upregulation of miR‐145 in the bones of AIS patients as well as the derived cultured bone cells. Strong positive correlation existed between miR‐145 and CTNNB1 (encoding β‐catenin) in primary osteoblasts, in which persistent activation of β‐catenin impaired osteocyte functions. Ectopic expression of miR‐145 induced β‐catenin expression whereas knockdown expression of miR‐145 decreased the active β‐catenin. Knockdown expression of miR‐145 also suppressed the formation of active β‐catenin/Tcf4 complex without influencing Tcf4 expression. Moreover, downregulating miR‐145 expression restored the normal expression of osteocyte markers, including osteoprotegerin (OPG), the transmembrane glycoprotein E11 and secretion of sclerostin (SOST) in AIS osteoblasts.

### 3.3 H19/miR‐675‐5p

Imbalance between two sides of paravertebral muscle is implicated in the onset and progression of scoliosis. Jiang and colleagues performed RNA sequencing to identify differentially expressed transcripts in the 5 pairs of paravertebral muscle of AIS cases.^51^ A total of 40 differentially expressed genes overrepresented in the peroxisome proliferator‐activated receptor (PPAR) signalling pathway and biosynthesis of unsaturated fatty acids were identified between the concave‐ and convex‐sided paravertebral muscles. Among the PPAR‐related genes, ADIPOQ, FABP4 and MSTN showed the most significant upregulation in concave‐sided muscle tissue. In contrast, H19, a lncRNA important for skeletal muscle differentiation and regeneration, was downregulated. The differential expression of ADIPOQ and H19 was further found to be correlated with earlier age of disease onset and larger spinal curve. Mechanistically, the authors found that miR‐675‐5p encoded by *H19* was an upstream negative regulator of the ADIPOQ expression as confirmed by negative correlation between miR‐675‐5p and ADIPOQ mRNA levels in muscle biopsies and the direct binding of miR‐675‐5p to the 3′‐untranslated region of ADIPOQ mRNA. Further upstream analysis revealed that differential CCCTC‐binding factor (CCTF) occupancy in the H19 imprinting control region was responsible the differential expression of H19. This study highlighted the potential involvement of the CCTF‐H19/miR‐675‐5p‐ADIPOQ axis in AIS development.

### 3.4 LncAIS

Abnormal osteogenic differentiation of mesenchymal stem cells (MSCs) is known to be associated with AIS. Zhang and colleagues performed microarray with bone marrow (BM)‐MSCs isolated from 12 AIS patients and 5 controls and identified 1483 deregulated lncRNAs—718 downregulated and 765 overexpressed.^52^ The reduction of the top downregulated lncRNA ENST00000453347 designated as lncAIS was further confirmed by RT‐qPCR in 30 AIS patients and 20 healthy controls. Functionally, knockdown of lncAIS inhibited osteogenic differentiation of BM‐MSCs in vitro and bone formation in vivo. Mechanistically, lncAIS was found to interact with the NF90 to enhance HOXD8 stability which promoted RUNX2 to induce osteogenic differentiation. This signalling cascade was impaired in AIS where lncAIS was downregulated. Collectively, these data supported the functional involvement of lncAIS in AIS pathogenesis.

### 3.5 SULT1C2A and miR‐466c‐5p

Our team has shown that embryonic expression of the lncRNA SULT1C2A was decreased whereas miR‐466c‐5p was upregulated in the rat model of vitamin A deficiency‐induced congenital scoliosis.^43^ Somitogenesis‐related genes, such as Foxo4, Sox9, Pax1 and Nkx3‐2, were downregulated on gestational day 9 upon maternal vitamin A deficiency. In this regard, reduced expression of SULT1C2A was found to increase the availability of miR‐466c‐5p and thereby decreasing Foxo4 expression. Spatiotemporal alterations of the SULT1C2A‐miR‐466c‐5p‐Foxo4 axis were paralleled by changes in phosphoinositide 3‐kinase (PI3K) expression and AKT phosphorylation. These data suggested that the aberrant downregulation of lncRNA SULT1C2A leads to reduced Foxo4 expression by increasing miR‐466c‐5p abundance in vitamin A deficiency‐associated congenital scoliosis.

### 3.6 miR‐15a

Li and colleagues studied the morphological changes, histone methylation alterations and cell growth‐related signalling pathway in the inferior facet joint cartilage in 10 control subjects and 11 patients with idiopathic scoliosis.^53^ Patients with idiopathic scoliosis showed increased number of proliferative chondrocytes as well as upregulation of BCL2 and COL2A1 in the facet joint cartilage, which was significantly narrowed. Furthermore, miR‐15a expression was decreased while the trimethylation of histone 3 lysine 9 (H3K9me3: a repressive chromatin mark) in promoter region of miR‐15a‐encoding gene was increased in the idiopathic scoliosis group. Mechanistically, suppressor of variegation 3‐9, drosophila homolog of 1 (SUV39H1) was found to be an upstream regulator that mediates epigenetic silencing of miR‐15a and thereby promoting BCL2 expression and proliferation of chondrogenic cells.

## 4 CONCLUSION AND FUTURE PERSPECTIVES

**Figure 1:**
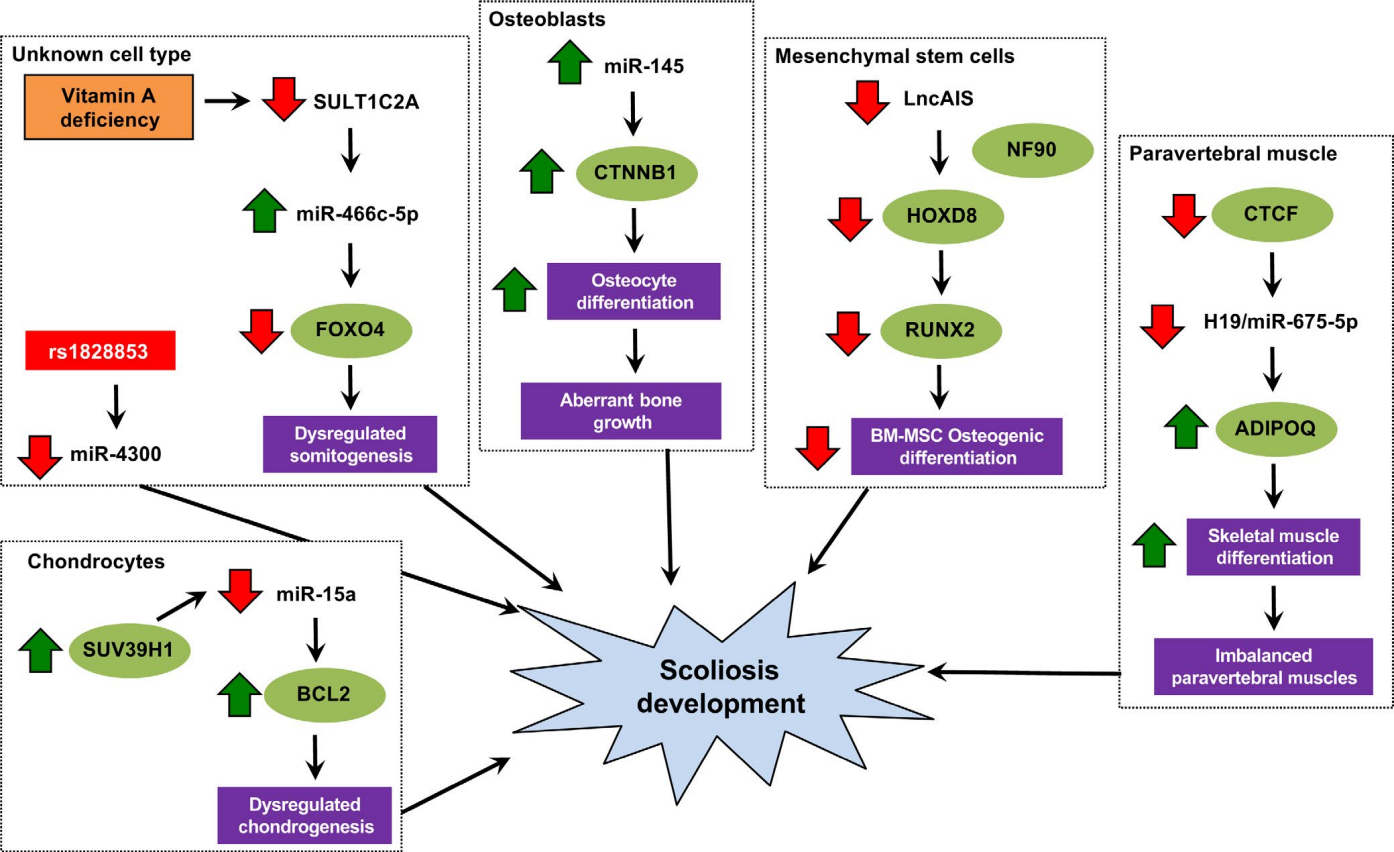
Pathogenic mechanism of deregulated ncRNAs in scoliosis

The cellular and molecular pathogenesis of scoliosis is complex, involves multiple aetiologies and depends on varying contributions of biomechanical, neuromuscular, developmental and biochemical abnormalities.^8^, ^29^ Among different subtypes of scoliosis, the ncRNA expression landscape has been relatively well depicted in AIS. Nevertheless, only few of the identified ncRNAs (ie miR‐145, miR‐675‐5p, LncAIS, SULT1C2A, miR‐466c‐5p, miR‐15a) have been functionally verified (Figure 1 and Table 2). These ncRNAs were found to primarily affect processes that are pertinent to the development of musculoskeletal system, including somitogenesis during embryo development, osteogenic differentiation of osteoblasts and bone marrow‐mesenchymal stem cells, paravertebral muscle differentiation and chondrogenesis. Future endeavours should therefore be put forth to characterize the functional roles of other deregulated ncRNAs in relevant cell line and animal models of scoliosis. The importance of ncRNAs should also be assessed in relation to clinicopathological parameters and treatment outcomes, so as to develop novel diagnostic and prognostic markers. In terms of treatment, it would be tantalizing to determine whether scoliosis progression could be prevented through restoring the expression of downregulated ncRNAs or silencing of the aberrantly upregulated ncRNAs. Nevertheless, the best way to deliver ncRNA‐directed therapeutics into specific tissue or cell types remain poorly defined. It is hopeful that, with more translational research in this area, ncRNAs will one day become part of our armamentarium to fight this potentially devastating disease.

**Table 2:** Functionally characterized ncRNAs in scoliosis

ncRNAs	Deregulation	Functional role	Related genes/loci	References
miR‐4300	SNPs	AIS risk progression	rs1828853	49
miR‐145	Upregulated	osteogenic differentiation	CTNNB1, TCF4	50
H19	Downregulated	skeletal muscle differentiation and regeneration	ADIPOQ	51
miR‐675‐5p	Downregulated	skeletal muscle differentiation and regeneration	ADIPOQ	51
LncAIS	Downregulated	Osteogenic differentiation, Bone formation	NF90 HOXD8 RUNX2	52
SULT1C2A	Downregulated	Somitogenesis	miR‐466c‐5p, FOXO4	43
miR‐466‐5c	Downregulated	Somitogenesis	FOXO4	43
miR‐15a	Downregulated	Chondrogenesis	BCL2	53

## CONFLICT OF INTEREST

There is no conflict of interest.

## AUTHOR CONTRIBUTIONS

Z L, XY L, JX S, LZ, M TV C and W KK W wrote the manuscript and searched the literature.

## References

[1] Li Z , Shen J , Qiu G , et al. Unplanned reoperation within 30 days of fusion surgery for spinal deformity. PLoS ONE. 2014;9(3):e87172. 24595145 10.1371/journal.pone.0087172 PMC3942308

[2] Lombardi G , Akoume MY , Colombini A , Moreau A , Banfi G . Biochemistry of adolescent idiopathic scoliosis. Adv Clin Chem. 2011;54:165‐182. 21874761 10.1016/b978-0-12-387025-4.00007-8

[3] Wai MG , Jun WW , Yee YA , et al. A review of pinealectomy‐induced melatonin‐deficient animal models for the study of etiopathogenesis of adolescent idiopathic scoliosis. Int J Mol Sci. 2014;15(9):16484‐16499. 25238413 10.3390/ijms150916484 PMC4200812

[4] Giampietro PF . Genetic aspects of congenital and idiopathic scoliosis. Scientifica. 2012;2012:152365. 24278672 10.6064/2012/152365 PMC3820596

[5] Wang S , Qiu Y , Ma Z , Xia C , Zhu F , Zhu Z . Expression of Runx2 and type X collagen in vertebral growth plate of patients with adolescent idiopathic scoliosis. Connect Tissue Res. 2010;51(3):188‐196. 20073986 10.3109/03008200903215590

[6] Shen J , Wang Z , Liu J , Xue X , Qiu G . Abnormalities associated with congenital scoliosis: a retrospective study of 226 Chinese surgical cases. Spine. 2013;38(10):814‐818. 23197014 10.1097/BRS.0b013e31827ed125

[7] Li Z , Shen J , Wu WK , et al. Vitamin A deficiency induces congenital spinal deformities in rats. PLoS ONE. 2012;7(10):e46565. 23071590 10.1371/journal.pone.0046565 PMC3465343

[8] Li Z , Yu X , Shen J . Environmental aspects of congenital scoliosis. Environ Sci Pollut Res Int. 2015;22(8):5751‐5755. 25628116 10.1007/s11356-015-4144-0

[9] Pourquie O . Vertebrate segmentation: from cyclic gene networks to scoliosis. Cell. 2011;145(5):650‐663. 21620133 10.1016/j.cell.2011.05.011 PMC3164975

[10] Sparrow DB , Chapman G , Smith AJ , et al. A mechanism for gene‐environment interaction in the etiology of congenital scoliosis. Cell. 2012;149(2):295‐306. 22484060 10.1016/j.cell.2012.02.054

[11] Man GCW , Wong JH , Wang WWJ , et al. Abnormal melatonin receptor 1B expression in osteoblasts from girls with adolescent idiopathic scoliosis. J Pineal Res. 2011;50(4):395‐402. 21480980 10.1111/j.1600-079X.2011.00857.x

[12] Hayes M , Gao X , Yu LX , et al. ptk7 mutant zebrafish models of congenital and idiopathic scoliosis implicate dysregulated Wnt signalling in disease. Nat Commun. 2014;5:4777. 25182715 10.1038/ncomms5777 PMC4155517

[13] Grauers A , Wang J , Einarsdottir E , et al. Candidate gene analysis and exome sequencing confirm LBX1 as a susceptibility gene for idiopathic scoliosis. Spine J. 2015;15(10):2239‐2246. 25987191 10.1016/j.spinee.2015.05.013

[14] Enjie X , Shao W , Jiang H , Lin T , Gao R , Zhou X . A genetic variant in GPR126 causing a decreased inclusion of exon 6 is associated with cartilage development in adolescent idiopathic scoliosis population. BioMed Res Int. 2019;4678969(10):1‐8. 10.1155/2019/4678969 PMC6388357 30886859

[15] Ogura Y , Kou I , Miura S , et al. A functional SNP in BNC2 is associated with adolescent idiopathic scoliosis. Am J Hum Genet. 2015;97(2):337‐342. 26211971 10.1016/j.ajhg.2015.06.012 PMC4573260

[16] Giampietro PF , Raggio CL , Reynolds CE , et al. An analysis of PAX1 in the development of vertebral malformations. Clin Genet. 2005;68(5):448‐453. 16207213 10.1111/j.1399-0004.2005.00520.x

[17] Veneziano D , Nigita G , Ferro A . Computational approaches for the analysis of ncRNA through deep sequencing techniques. Front Bioeng Biotechnol. 2015;3:77. 26090362 10.3389/fbioe.2015.00077 PMC4453482

[18] Place RF , Noonan EJ . Non‐coding RNAs turn up the heat: an emerging layer of novel regulators in the mammalian heat shock response. Cell Stress Chaperones. 2014;19(2):159‐172. 24002685 10.1007/s12192-013-0456-5 PMC3933615

[19] Li T , Mo X , Fu L , Xiao B , Guo J . Molecular mechanisms of long noncoding RNAs on gastric cancer. Oncotarget. 2016;7(8):8601‐8612. 26788991 10.18632/oncotarget.6926 PMC4890990

[20] Li Z , Lei H , Luo M , et al. DNA methylation downregulated mir‐10b acts as a tumor suppressor in gastric cancer. Gastric Cancer. 2015;18(1):43‐54. 24481854 10.1007/s10120-014-0340-8

[21] Wu WKK , Law PTY , Lee CW , et al. MicroRNA in colorectal cancer: from benchtop to bedside. Carcinogenesis. 2011;32(3):247‐253. 21081475 10.1093/carcin/bgq243

[22] Marchese FP , Raimondi I , Huarte M . The multidimensional mechanisms of long noncoding RNA function. Genome Biol. 2017;18(1):206. 29084573 10.1186/s13059-017-1348-2 PMC5663108

[23] Barrett SP , Salzman J . Circular RNAs: analysis, expression and potential functions. Development (Cambridge, England). 2016;143(11):1838‐1847. 10.1242/dev.128074 PMC4920157 27246710

[24] Yu X , Li Z , Chen G , Wu WK . MicroRNA‐10b induces vascular muscle cell proliferation through Akt pathway by targeting TIP30. Curr Vasc Pharmacol. 2015;13(5):679‐686. 25612666 10.2174/1570161113666150123112751

[25] Li Z , Shen JX , Chan MTV , Wu WKK . MicroRNA‐379 suppresses osteosarcoma progression by targeting PDK1. J Cell Mol Med. 2017;21(2):315‐323. 27781416 10.1111/jcmm.12966 PMC5264134

[26] Li Z , Li XY , Chen X , et al. Emerging roles of long non‐coding RNAs in neuropathic pain. Cell Prolif. 2019;52(1):e12528. 30362191 10.1111/cpr.12528 PMC6430490

[27] Li Z , Li XY , Chen C , et al. Long non‐coding RNAs in nucleus pulposus cell function and intervertebral disc degeneration. Cell Prolif. 2018;51(5):e12483. 30039593 10.1111/cpr.12483 PMC6528936

[28] Li Z , Yu X , Shen JX . Long non‐coding RNAs: emerging players in osteosarcoma. Tumor Biol. 2016;37(3):2811‐2816. 10.1007/s13277-015-4749-4 26718212

[29] Zheng JL , Yi D , Liu Y , Wang MQ , Zhu YL , Shi HZ . Long nonding RNA UCA1 regulates neural stem cell differentiation by controlling miR‐1/Hes1 expression. Am J Transl Res. 2017;9(8):3696‐3704. 28861160 PMC5575183

[30] Zhao J , Gao Z , Zhang C , Wu H , Gu R , Jiang R . Long non‐coding RNA ASBEL promotes osteosarcoma cell proliferation, migration and invasion by regulating microRNA‐21. J Cell Biochem. 2018;119(8):6461‐6469. 29323740 10.1002/jcb.26671

[31] Yu Y , Yang J , Li Q , Xu B , Lian Y , Miao L . LINC00152: A pivotal oncogenic long non‐coding RNA in human cancers. Cell Prolif. 2017;50(4):e12349. 10.1111/cpr.12349 PMC6529135 28464433

[32] Zhang JM , Yin MN , Peng G , Zhao YC . CRNDE: an important oncogenic long non‐coding RNA in human cancers. Cell Prolif. 2018;51(3):e12440. 29405523 10.1111/cpr.12440 PMC6528921

[33] Yu XJ , Zou LH , Jin JH , et al. Long noncoding RNAs and novel inflammatory genes determined by RNA sequencing in human lymphocytes are up‐regulated in permanent atrial fibrillation. Am J Transl Res. 2017;9(5):2314‐2326. 28559982 PMC5446514

[34] Wang XB , Lv GH , Li J , Wang B , Zhang QS , Lu C . LncRNA‐RP11‐296A18.3/miR‐138/HIF1A pathway regulates the proliferation ECM synthesis of human nucleus pulposus cells (HNPCs). J Cell Biochem. 2017;118(12):4862‐4871. 28543639 10.1002/jcb.26166

[35] Bochenek G , Hasler R , El Mokhtari NE , et al. The large non‐coding RNA ANRIL, which is associated with atherosclerosis, periodontitis and several forms of cancer, regulates ADIPOR1, VAMP3 and C11ORF10. Hum Mol Genet. 2013;22(22):4516‐4527. 23813974 10.1093/hmg/ddt299

[36] Xie Z , Chen G , Zhang X , et al. Salivary microRNAs as promising biomarkers for detection of esophageal cancer. PLoS ONE. 2013;8(4):e57502. 23560033 10.1371/journal.pone.0057502 PMC3613402

[37] Zhu W , Zhou K , Zha Y , et al. Diagnostic value of serum miR‐182, miR‐183, miR‐210, and miR‐126 levels in patients with early‐stage non‐small cell lung cancer. PLoS ONE. 2016;11(4):e0153046. 27093275 10.1371/journal.pone.0153046 PMC4836744

[38] Shimizu T , Suzuki H , Nojima M , et al. Methylation of a panel of microRNA genes is a novel biomarker for detection of bladder cancer. Eur Urol. 2013;63(6):1091‐1100. 23200812 10.1016/j.eururo.2012.11.030

[39] Yu X , Li Z , Shen J , et al. MicroRNA‐10b promotes nucleus pulposus cell proliferation through RhoC‐Akt pathway by targeting HOXD10 in intervetebral disc degeneration. PLoS ONE. 2013;8(12):e83080. 24376640 10.1371/journal.pone.0083080 PMC3869743

[40] Yamasaki K , Nakasa T , Miyaki S , Yamasaki T , Yasunaga Y , Ochi M . Angiogenic microRNA‐210 is present in cells surrounding osteonecrosis. J Orthop Res. 2012;30(8):1263‐1270. 22287106 10.1002/jor.22079

[41] Chen WK , Yu XH , Yang W , et al. IncRNAs: novel players in intervertebral disc degeneration and osteoarthritis. Cell Prolif. 2017;50(1):e12313. 10.1111/cpr.12313 PMC6529103 27859817

[42] Chen C , Tan HN , Bi JQ , et al. Identification of competing endogenous RNA regulatory networks in vitamin a deficiency‐induced congenital scoliosis by transcriptome sequencing analysis. Cell Physiol Biochem. 2018;48(5):2134‐2146. 30110682 10.1159/000492556

[43] Chen C , Tan H , Bi J , et al. LncRNA‐SULT1C2A regulates Foxo4 in congenital scoliosis by targeting rno‐miR‐466c‐5p through PI3K‐ATK signalling. J Cell Mol Med. 2019;23(7):4582‐4591. 31044535 10.1111/jcmm.14355 PMC6584475

[44] Tong J , Zhao W , Lv H , Li W , Chen Z , Zhang C . Transcriptomic profiling in human decidua of severe preeclampsia detected by RNA sequencing. J Cell Biochem. 2018;119(1):607‐615. 28618048 10.1002/jcb.26221

[45] Zhou J , Fan Y , Chen H . Analyses of long non‐coding RNA and mRNA profiles in the spinal cord of rats using RNA sequencing during the progression of neuropathic pain in an SNI model. RNA Biol. 2017;14(12):1810‐1826. 28854101 10.1080/15476286.2017.1371400 PMC5731818

[46] Xiao‐Yang L , Liang W , Bin Y , Qian‐Yu Z , Yi‐Peng W . Expression signatures of long noncoding RNAs in adolescent idiopathic scoliosis. BioMed Res Int. 2015;2015:276049. 26421281 10.1155/2015/276049 PMC4569756

[47] García‐Giménez JL , Rubio‐Belmar PA , Peiró‐Chova L , et al. Circulating miRNAs as diagnostic biomarkers for adolescent idiopathic scoliosis. Sci Rep. 2018;8(1):2646. 29422531 10.1038/s41598-018-21146-x PMC5805715

[48] Seco‐Cervera M , González‐Rodríguez D , Ibáñez‐Cabellos JS , Peiró‐Chova L , Pallardó FV , García‐Giménez JL . Small RNA‐seq analysis of circulating miRNAs to identify phenotypic variability in Friedreich's ataxia patients. Sci Data. 2018;5:180021. 29509186 10.1038/sdata.2018.21 PMC5839159

[49] Ogura Y , Kou I , Takahashi Y , et al. A functional variant in MIR4300HG, the host gene of microRNA MIR4300 is associated with progression of adolescent idiopathic scoliosis. Hum Mol Genet. 2017;26(20):4086‐4092. 29016859 10.1093/hmg/ddx291

[50] Jiajun Z , Huanxiong C , Ross KKL , et al. Aberrant miR‐145‐5p/β‐catenin signal impairs osteocyte function in adolescent idiopathic scoliosis. FASEB J. 2018;32 10.1096/fj.201800281. [Epub ahead of print]. 29906249

[51] Jiang H , Yang F , Lin T , et al. Asymmetric expression of H19 and ADIPOQ in concave/convex paravertebral muscles is associated with severe adolescent idiopathic scoliosis. Mol Med. 2018;24(1):48. 30241458 10.1186/s10020-018-0049-y PMC6145194

[52] Zhuang Q , Ye B , Hui S , et al. Long noncoding RNA lncAIS downregulation in mesenchymal stem cells is implicated in the pathogenesis of adolescent idiopathic scoliosis. Cell Death Differ. 2019;26(9):1700‐1715. 30464226 10.1038/s41418-018-0240-2 PMC6748078

[53] Li J , Yang G , Liu S , Wang L , Liang Z , Zhang H . Suv39h1 promotes facet joint chondrocyte proliferation by targeting miR‐15a/Bcl2 in idiopathic scoliosis patients. Clin Epigenetics. 2019;11(1):107. 31337422 10.1186/s13148-019-0706-1 PMC6651996

